# Supplementary material for: Are carbon emissions trading and green financial instruments synergistic? -Comprehensive quantitative research based on content analysis
Source: PLoS One. 2024 Mar 7;19(3):e0298601. doi: 10.1371/journal.pone.0298601 (PMC10919601; doi:10.1371/journal.pone.0298601)
Supplement: S1 Data — (ZIP) [file pone.0298601.s001.zip › Data/地级市Z_F工作报告.zip!/║ú─╧/║ú┐┌╩╨/└┤╫╘╣1⁄2╓┌║┼í╛┬φ┐╦╩2╛▌═°í┐_2018.docx]

各位代表：

现在，我代表海口市人民政府向大会报告工作，请予审议，并请各位政协委员和列席人员提出意见。

2017年工作回顾

一年来，我们坚持以习近平新时代中国特色社会主义思想为指导，认真贯彻落实党的十八大和十九大精神，在省委省政府和市委的坚强领导下，在市人大、市政协的监督支持下，坚持稳中求进工作总基调，高质量推进国际化滨江滨海花园城市建设，统筹抓好稳增长、促改革、调结构、惠民生、防风险等各项工作，较好完成了市第十六届人民代表大会第二次会议确定的目标任务，成功创建全国文明城市和国家卫生城市，实现了本届政府的良好开局。全年实现地区生产总值1390亿元，同比增长7.5%；固定资产投资1415.5亿元，增长11.3%；全口径一般公共预算收入388.5亿元，增长16.6%，其中地方一般公共预算收入125.4亿元，增长12.8%；社会消费品零售总额726亿元，增长11%；城乡常住居民人均可支配收入28701元，增长8.5%；居民消费价格指数上涨3.3%。

一年来，主要抓了以下六个方面的工作：

（一）以决战决胜之势，成功摘得全国文明城市和国家卫生城市“金字招牌”

城市规划优化提升。搭建“多规合一”[1]综合信息平台，启动新一轮城市总体规划修编。科学制定城市更新规划和行动纲要，海甸溪两岸综合整治、骑楼老街改造等首批城市更新示范项目加快实施。“五网”[2]综合规划、城市轨道交通线网规划和新海生态临港新城概念规划基本完成。滨江滨海、海绵城市[3]、湿地和郊野公园等专项规划加快推进。入选全国第二批城市设计试点和第三批生态修复城市修补试点。

城市建设有序推进。持续加大“五化”[4]力度，建立健全环卫监督净化考核机制，建成区主要道路机扫率达100%；滨海大道、国兴大道等主要道路和西海岸带状公园、万绿园等23个门户景观完成绿化提升；实施三角梅储备和控花工程，龙昆南路等城市主干道完成彩化；海口湾片区235栋建筑楼宇和滨海大道、火车站等重点路段和区域完成亮化改造；外立面改造、广告牌整治等美化工作稳步推进。省重点项目完成投资691亿元，占年度计划的108%。美兰机场二期、绕城高速改扩建、南渡江引水工程等“五网”项目加快推进，市民游客中心开工建设，汽车客运总站投入试运行，地下综合管廊建成38.5公里。累计打通“断头路”64条，改造主干道路93条、小街小巷2603条、农贸市场46家、城区积水点24处，打造15分钟便民生活圈46处，筑牢了城市的“里子”。

城市管理明显改善。智慧海口加快建设，“12345”联动平台+“椰城市民云”“椰城政务云”“椰城创新云”[5-7]的智慧城市框架基本形成。“椰城市民云”整合38个部门、139项公共服务事项，为市民提供全生命周期的大数据服务；“12345”智慧联动平台发挥“指挥棒”“绣花针”“连心桥”作用，推行区（局）长值班制，探索建立综合行政执法体制，创新实施“12345+网格化”和30分钟快速响应处置机制，日均接话量增长6.1倍，群众满意率从48%提高到95%，获全国政府服务热线“先锋奖”“骏马奖”。

城市文明日益彰显。社会主义核心价值观和优秀传统文化广泛弘扬，《永远的更路簿》获国家“五个一工程”[8]奖，《原始黎陶》《椰壳新生》等非物质文化遗产纪录片在央视播出。大坡镇和斌腾村、美孝村、冯塘村等获评全国文明村镇。立法设立“全民公益日”，全市注册志愿者超过26万人，入选中国“志愿之城”试点城市。凡人善举层出不穷，11人获得全国道德模范提名奖、21人入选中国好人榜，甘当积水警示牌的“守坑叔”、街头抢救老人的“好医生”、生命最后一瞬停稳公交车的“好司机”等一大批最美人物的先进事迹传遍琼州大地，得到社会各界和中央等各级媒体的广泛赞誉，文明风尚正成为全体市民的自觉行动和价值追求，见贤思齐、崇德向善的社会氛围更加浓厚，城市变得更有温度、更加温馨。

各位代表，全市上下众志成城、勠力同心，经过艰苦卓绝的接续奋斗，成功创建了全国文明城市和国家卫生城市，这是我们贯彻落实习近平新时代中国特色社会主义思想和习近平总书记2013年视察海南重要讲话精神的重大成果，是海南建省办经济特区30年来文明城市“零的突破”，也是海口建设国际化滨江滨海花园城市的重要里程碑，凝聚起全市人民团结奋进的磅礴力量，为我们奋力走好新时代的长征路注入了强大动力。

（二）深入推进供给侧结构性改革，产业结构不断优化

全市十二个重点产业增加值占全省的35.9%，其中6个产业占全省比重超过50%，发展的质量和效益不断提高。全域旅游扎实推进，“美丽海南百千工程”[9]加快建设，镇墟旅游化改造有序开展，施茶村等26个村入选海南美丽乡村；成立旅游警察支队，美兰机场旅客吞吐量达2258万人次，创历史新高；全市接待游客2428万人次，旅游总收入266亿元，分别增长11.1%和14.3%，获评全国厕所革命先进市。热带高效农业加快发展，成功申报实施“丝路海口?田园综合体”[10]和农村产业融合发展试点等国家级项目，石山黑豆、石山壅羊获国家地理标志证明商标，桂林洋国家热带农业公园一期开园。房地产业平稳健康发展，落实和深化“两个暂停”[11]政策，出台装配式建筑[12]发展政策。互联网产业蓬勃发展，营业收入205亿元，增长32.3%；滨海国际电子商务园区加快建设，复兴城互联网创新创业园、江东电子商务园获评国家级示范基地，海口入选2017中国“互联网+”数字经济总指数百强城市。医疗健康产业稳步推进，上海六院海口骨科与糖尿病医院、上海中医药大学附属岳阳医院海口分院开诊，观澜湖中医国际康养中心投入运营。金融业不断壮大，民生银行和5家证券公司落户，浦发银行创新开展离岸金融业务，全年实现增加值167亿元，增长9.8%。会展业快速发展，举办上规模会展活动270场，综合收入突破100亿元，分别增长27%和25%，荣获中国会议产业大会“会奖之星”。海洋产业创新发展，获批国家海洋经济创新发展示范城市。现代物流业加快推进，马村港区三期散货码头、新海港区二期建成投产，秀英港区滚装轮渡业务完成搬迁；海口港集装箱吞吐量183万标箱，增长30.6%。医药产业集群初步成形，出台鼓励药企开展“一致性评价”[13]的政策，全年实现医药产值191.6亿元，增长19.3%，占全省98%。低碳制造产业加快培育，新增高新技术企业64家，总数达207家，占全省77%；新增12家院士工作站，灵康制药、椰国食品、一汽海马等3家企业获中国专利奖；获评国家知识产权试点城市先进集体。文体产业发展提速，中国足球（南方）训练基地、国家帆船帆板训练基地、五源河文体中心加快建设，《芳华》等4部影视作品在我市完成拍摄并上映，大大提升了海口旅游品牌。

（三）深化改革开放，发展的内生动力持续增强

重点领域改革深入推进，“多规合一”“放管服”[14]等改革持续发力，实施“一窗受理、集成服务”[15]，在“五网”基础设施建设中全面推行“极简审批”[16]，“不见面审批”[17]达84.5%，提前一年完成省政府任务要求。综保区推出40项复制自贸区改革试点经验清单，高新区入选国家创业创新示范基地。全面推行“多证合一”[18]，全年新增市场主体5.1万个，占全省四成。国资国企改革稳步推进，完成国有农场公司化改革和社会职能属地化管理。在全省首批通过农村土地承包经营权确权登记颁证成果验收，农村集体产权制度改革琼山试点得到国家农业部肯定。

对外开放不断扩大。推进“一带一路”互联互通建设，新增外贸集装箱班轮航线3条，新开通境外定期直飞航线12条，开通海口至越南、菲律宾邮轮旅游航线。在境外12个国家（地区）举办旅游促销活动，全年接待入境游客18.2万人次，同比增长33.3%。服务贸易创新试点稳步推进，跨境电商产业园建成使用。全年实现外贸出口55.5亿元，同比增长6.4%。与菲律宾塔贡市、印尼北龙目市结好，国际友城达到35个，遍布全球5大洲29个国家。

区域合作全面加速。积极推进琼州海峡经济带建设，湛江—海口高铁、铁路物流等前期规划加紧推进；与湛江建立全方位常态化沟通联席机制，以港航一体化为先导，两岸直升机通航运行，水上飞机试飞成功，客滚快船加快推进。铺前大桥、江东大道二期等“海澄文”[19]基础设施联通项目加快建设。坚持“用最好的资源吸引最好的投资”，华侨城等一批大型央企和民企落户海口，全年引进项目141个、签约金额1781亿元，注册落地率79%。

（四）不断强化生态环境保护，生态文明建设成效明显

认真抓好中央环保督察和国家海洋督察反馈问题整改，严管重罚，立案查处环境违法案件530宗，制定防治汽车鸣笛和远光灯违法行为、环境违法行为有奖举报等11个管理办法，出台《海口市扬尘污染防治办法》《海口市美舍河保护管理规定》《关于加强湿地保护管理的决定》，在秀英区开展自然资源资产责任审计试点。扎实开展“六大专项整治”[20]，城市、农村生活垃圾无害化处理率分别达100%、95%以上，全市中型以上餐饮企业油烟净化设施安装率达90%以上，出租车、公交车的清洁能源使用率分别达100%、83%以上，空气质量连续五年在全国74个重点城市中排名第一。拆除违法建筑212万平方米、防控违法建筑73万平方米。在全省率先推行“河长制”，对32个水体进行综合治理，美舍河“控源截污、内源治理、生态修复、景观提升”治理模式在全省推广，获批国家级水利风景区。海口入围全国首批“湾长制”试点城市，实现“河湾同治”。民航航线俯视区等修复工作稳步推进，湿地保护修复的做法在全国林业系统推广，五源河湿地公园、美舍河湿地公园获评国家湿地公园试点，潭丰洋等5个省级湿地公园加快建设，获首批国际湿地城市认证提名。

（五）以人民为中心，发展成果不断惠及全市百姓

全年民生支出139.7亿元，占地方一般公共预算支出的70.4%，获评“中国十大幸福城市”和全国“惠民服务优秀城市”省会十强。引进“好校长、好教师”12名，北大附中附小海口学校加快建设，北师大海口附校、海景学校建成招生，新增学位9500个。全年城镇新增就业3.5万人，组织农村富余劳动力转移就业7300人，年末城镇登记失业率1.3%。新增社保扩面6.6万人，发放低保和特困救助金1.2亿元，惠及困难群众2.6万人。新一轮省市财政体制改革拉平了海口与其他市县共享税分成比例，为改善民生增强了财力保障。深入推进脱贫攻坚，完成建档立卡贫困人口629户2773人的减贫任务，5个村退出贫困村行列。成功引进一批岛外优质医疗资源，新增“微信”“支付宝”“椰城市民云”等网上预约诊疗、挂号、结算等便民措施，所有公立医疗机构实施住院“先看病、后付费”，累计垫付费用4.1亿元、受益群众7.1万人；6家公立医院全面取消药品加成，为群众节省费用4128万元；成功创建国家流动人口卫生计生基本公共服务示范市。完成城市棚户区改造2.3万户，占省下达任务的121.8%；改造农村危房400户，全省考核第一。让公交回归民生本位，基本完成全市公交运营主体整合，优化调整公交线网，总线路从96条增加至152条，37条公交线路实现实时位置可查询。狠抓“菜篮子”工程，坚持“基本菜”“一元菜”等惠民措施，15种蔬菜平均零售价同比下降17.7%，在全国36个大中城市和全省各市县的菜价排名“双下降”。启动“放心粮油”工程建设，粮油市场供需双稳。食品药品安全状况明显提升，得到国务院督察组高度肯定。圆满完成禁毒三年大会战第一阶段工作，破获刑事案件同比上升4.5%、立案数下降23%，获评第二届全国创新社会治理城市，龙华蝉联全国综治“长安杯”；全市安全生产形势整体稳定，全省考核第一；获评全国地市级防震减灾工作综合考核先进单位。

（六）坚持实干担当，政府自身建设进一步加强

各级政府认真学习宣传贯彻习近平新时代中国特色社会主义思想和党的十九大精神，扎实推进“两学一做”学习教育常态化制度化，深入开展“大研讨大行动”，实施重点课题研究71项。严格落实中央八项规定、省委省政府二十条规定和市委二十一条规定，坚决履行党风廉政建设“一岗双责”[21]，建立巡察与审计协作机制，支持纪检监察部门依纪依法查处腐败案件，立案462件，处分465人，为发展提供了坚强的政治保证和纪律保证。自觉接受人大法律监督、工作监督和政协民主监督，认真办理人大代表建议484件、政协委员提案654件，办结率为100%。强化政府立法工作，提请市人大常委会审议法规议案4件，修改政府规章1件、废止13件，依法行政工作全省考核第一。全市各级各部门牢固树立“四个意识”，工作夜以继日，坚持“钉钉子”、马上就办、办就办好，锤炼了一支吃苦耐劳能打硬仗的干部队伍，确保了党中央国务院、省委省政府和市委各项决策部署得到有效落实。

此外，统计荣获全国先进单位，人防、气象、三防、保密、档案、史志、科协、工商联、民族宗教、外事侨务、海防口岸、对台事务等工作取得新进展，工会、青少年、妇女儿童、老龄、残疾人、慈善等事业取得新进步。

各位代表！2017年是很不平凡的一年，成绩来之不易，这是党中央国务院、省委省政府和市委坚强领导的结果，是市人大、市政协及各位代表、各位委员支持的结果，是全市224万人民群众攻坚克难、共同奋斗的结果。在这里，我谨代表市人民政府，向全市人民，向人大代表和政协委员，向各民主党派、工商联和社会各界人士，向驻市部队、武警官兵和公安司法干警，向关心支持海口的广大海外侨胞、港澳台同胞，表示崇高的敬意和衷心的感谢！

在肯定成绩的同时，必须清醒看到，我们的工作与人民日益增长的美好生活需要还有差距，发展不平衡不充分的问题突出。经济总量小，实体经济不强，城乡居民收入不高，发展质量和效益有待提升；交通领域短板突出，教育、医疗等方面还需持续发力，镇墟和农村基础设施和公共服务投入不足；生态环境保护任重道远，社会文明水平有待进一步提高；对外开放、国际化水平还不够高，人才资源相对匮乏，体制机制活力有待进一步释放；营商环境有待进一步改善，群众和企业仍受证照证明、审批环节过多之累和办事慢、办事难之苦；一些公职人员的庸懒散奢和吃拿卡要等行为屡禁不止。这些问题都需要我们认真加以整改。

2018年工作安排

今年是我们贯彻落实党的十九大精神的开局之年，是全国改革开放40周年、海南建省办经济特区30周年，是习近平总书记视察海南发表重要讲话5周年，也是海口决胜全面建成小康社会、实施“十三五”规划承上启下的关键一年。

根据市委部署，今年政府工作的总体要求是：高举中国特色社会主义伟大旗帜，全面贯彻党的十九大精神，以习近平新时代中国特色社会主义思想为指导，深入贯彻习近平总书记2013年视察海南重要讲话精神，落实省第七次党代会、省委七届历次全会和市第十三次党代会、市委十三届历次全会的决策部署，坚持稳中求进工作总基调，践行新发展理念，按照高质量发展要求，统筹推进“五位一体”总体布局和协调推进“四个全面”战略布局，坚持以供给侧结构性改革为主线，突出抓重点、补短板、强弱项，坚决打好防范化解重大风险、精准脱贫、污染防治“三大”攻坚战，加快以“五化”先行的城市更新工作，深入实施乡村振兴战略，做大做优做强海口、建设国际化滨江滨海花园城市，坚决扛起建设美好新海南的省会担当。

主要预期目标是：全市生产总值增长7.2%左右，固定资产投资增长10%左右，社会消费品零售总额增长10%左右，地方一般公共预算收入增长10%左右，城乡居民收入增长8%左右，居民消费价格涨幅控制在3.8%左右，城镇登记失业率控制在3%以内，全面完成省下达的节能减排降碳控制目标。

围绕上述目标，我们将着力抓好以下五个方面、20项工作。

（一）提高经济发展质量和效益，着力打造经济繁荣海口

坚持质量第一、效益优先，通过质量变革、效率变革、动力变革，培育新增长极、形成新动能，做大做强实体经济，逐步构建具有海口特色的现代化经济体系。

1.深入推进供给侧结构性改革。加快重点产业成形成势，力争十二个重点产业占GDP比重超过80%，占全省比重提高两个百分点。“点线面”推动全域旅游，长影环球100试营业，启动桂林洋国家热带农业公园研创中心、展销中心建设，力争年内建成国家全域旅游示范区。大力实施“旅游+”工程，加快培育和发展邮轮旅游、房车自驾游、低空飞行等旅游新业态。制定鼓励出入境旅客海口中转和邮轮旅游的扶持政策，力争入境游客增长15%。加快互联网产业发展，推进复兴城互联网创新创业园建设，实现互联网产业营业收入300亿元以上。继续深化与岛外优质医疗健康资源合作，推进上海第一妇婴保健院合作项目落地。大力发展现代金融和总部经济，争取引进新型金融业态机构、配套服务机构落户海口，积极申报第二批绿色金融示范区。抓好大型会展场馆建设，引进和培育一批会展和会议品牌。推进国家海洋经济创新发展示范城市建设，加快海洋生物产业、海洋高端装备等示范项目建设。加快大型物流园区、配套完善分拨中心以及货运枢纽建设，完善机场、港口等物流设施和集疏运体系。做大做强医药产业，加快医疗器械、生物医药和海洋生物制药布局，引进国内外龙头医药企业、研究机构，全力争取国家重大新药创制成果转移转化试点，力争医药产业产值突破200亿元。积极培育发展影视制作、动漫游戏等文体产业，吸引国内外顶级文化平台和体育赛事落户。坚持“房子是用来住的，不是用来炒的”定位，保障性需求由政府统筹，改善性需求由市场调节，投资性需求靠制度限制，促进房地产市场持续平稳健康发展。

当前海口进入了高质量发展的转型攻坚期，我们要通过盘活挖潜、提质增效、改革创新、优化营商环境“四大举措”，盘活闲置和低效开发土地、盘活签约项目落地开工、盘活存量楼宇厂房、盘活国企国资，制定三年行动计划，在产业、民生、基础设施等领域谋划一批大项目，同时研究出台鼓励总部企业聚集发展的政策措施，积极引进一批跨国公司、行业龙头企业在海口设立区域总部，推动海口经济持续健康发展。

2.全面强化创新驱动。落实人才新政，围绕重点产业和战略性新兴产业，开展高层次人才、紧缺人才引进计划，开通高校青年引才直通车。在企业和人员落户、人才激励、办公补助、出入境与通关、子女教育、住房保障等方面出台政策，吸引更多企业和优秀人才。加强国家重大科技专项对接，争取更多科研院所、国家重点实验室、院士工作站落户。重点招商引进和培育一批创新型企业，力争今年新增高新技术企业30家以上。鼓励企业创建产学研创新联盟，强化知识产权创造、保护和运用，争取每万人有效发明专利拥有量达7件以上。加快推进创新型城市建设，引导全社会加大科技研发投入。

3.大力实施乡村振兴战略。按照产业兴旺、生态宜居、乡风文明、治理有效、生活富裕的总要求，加快推进农业农村现代化。大力发展热带特色高效农业，围绕“一镇一业”“一村一品”，培育壮大海口特色农业品牌。大力发展农产品电子商务，争取海南农产品拍卖交易中心落户海口。加快推进一二三次产业融合发展，出台鼓励农产品深加工、田园综合体、乡村民宿、共享农庄发展扶持政策。推进“美丽海南百千工程”，加快演丰、石山等10个特色产业小镇建设，打造一批美丽乡村示范点。加强农村房屋建设规划报建和宅基地管理，改造农村危房1600户，加快推进农村公路、农田水利等“五网”基础设施建设，全面完成自然村通硬化路等六大类工程[22]960公里。启动农村生活污水处理设施建设三年行动计划，完成农村改厕7400户。推进农村集体产权制度改革，完成琼山区农村集体产权制度改革国家级试点验收和推广。引导规范农村土地流转，恢复撂荒地生产6000亩以上。鼓励和支持农民工、高校毕业生和退役士兵等各类人员返乡下乡创业，积极培育新型农业经营主体，培养造就一支懂农业、爱农村、爱农民的“三农”工作队伍。千方百计提高农民收入，确保农民人均可支配收入增长8%以上。

4.加快“五网”基础设施建设。加快构建“一江两‘岸’、东西双港驱动、南北协调发展”城市格局。按照“一枢纽、两地、三中心”[23]定位，加快新海生态临港新城建设，上半年开工建设新海港区客运枢纽；高标准推进滨江规划建设，加快美兰机场二期扩建、马村港区三期四期、新海港区三期等重大交通工程建设，推进疏港货运快速干道、海秀快速路二期、海甸溪和南渡江下穿通道等工程。统筹建设地上地下市政公用设施，加快主城区地下通信管网建设，实现光纤宽带网络和4G信号全覆盖。加快推进城市积水点改造，完善雨污分流管网配套，力争三年内解决城市内涝问题。基本完成南渡江引水工程建设，开工建设永庄十万吨水厂三期、江东十万吨水厂及配套管网。全力推进电网供电保障和抗灾能力三年行动计划，实现主城区年户均停电时间降至4个小时以内，达到全国一流水平。

5.持续深化重点领域改革。着力优化营商环境，不断激发市场活力和社会创造力。深化行政审批制度改革，围绕市民游客和企业反映的痛点和难点问题，针对市场准入、施工许可、服务贸易等审批办理制定专项行动计划，进一步清理行政事业性收费，减轻企业负担。加快培育和引进一批高水平的会计、审计、法律、仲裁、咨询、检验检测、评估等专业服务机构。“不见面审批”实现100%上线，所有市场主体的各项登记业务实现全程电子化。探索实施“证照分离”改革[24]，力争全年新增市场主体4万个以上。完善促进消费的体制机制，增强消费对经济发展的基础性作用。统筹推进财政体制机制改革，合理划分市、区事权和支出责任，强化对四个区和三个开发区绩效考核。健全“多规合一”“编、审、管、监”分离的规划管理机制，调整完善市、区城乡规划管理体制，加强城乡规划集中统一管理。推进“拨改投”改革[25]，引导民间资本进入更多的领域。完善债务风险预警机制，以经营城市的理念加大政府存量债务化解和处置力度。推进国有企业混合制改革，积极引进国内外战略资本和行业龙头企业参与我市国有企业改革，引导和支持本地企业上市。继续深化医药卫生、教育、农场管理体制机制等各项改革。

6.努力构建全方位开放格局。积极落实海南参与“一带一路”行动计划，推进泛南海经济圈建设，新开通境外空中航线8条以上，开通海口至新加坡、马来西亚集装箱班轮航线，实现与东盟沿海国家集装箱航线全覆盖。以港通路通为纽带，深化泛珠三角区域合作，积极融入粤港澳大湾区、北部湾城市群建设；加强琼州海峡经济带建设，继续推进琼州海峡港航一体化，年内开通高速客滚船，积极推动湛江—海口高铁规划建设，谋划开通湛江、北海、钦州等地的水上飞机线路，打造琼州海峡“半小时立体交通圈”。加快“海澄文”一体化进程，积极推动琼北滨海旅游公路、绕城高速二期等互联互通项目规划建设，加快推进与文昌清澜港、临高金牌港等公共码头资源整合。发展跨境电商外贸新业态，积极申报海口跨境电商综合试验区，推进国际购物中心规划建设。

（二）加强和创新社会治理，着力打造社会文明海口

秉承“城市管理应该像绣花一样精细”理念，科学地规划、合规地建设、绿色地发展、优化地管理、贴心地服务，建立持续巩固提升“创文创卫”[26]的长效工作机制，统筹全市社会文明和文化事业各方面建设。

7.加强精细化治理的智慧城市建设。不断强化“12345”智慧联动平台“指挥棒”“绣花针”“连心桥”的作用，充分利用“椰城市民云”“椰城政务云”“椰城创新云”等先进技术手段和功能，建立跨部门、跨行业、跨层级的大数据共享和开发利用机制，为市民游客和企业提供便捷、实用的信息服务。继续推行“12345+网格化”，强化各区各部门一体化联动，努力构建快速响应诉求、一线化解矛盾的联动格局，不断提升城市治理水平和服务质量。启动交通治堵三年行动计划，开展轨道交通前期工作和环岛高铁海口站—美兰站城际快线化改造，优化主次干道路、重要支路和过街通道建设，打通断头路10条；实施公交优先战略，加强公交专用道建设，继续优化公交线路，年内实现智能电子站牌全覆盖；加快智慧交通建设，实施“路长制”，优化交通组织，鼓励和引导市民绿色出行。健全市、区、街、社区四级联动物业管理体制机制，修订完善市物业管理办法。提升和完善“三无”小区[27]消防设施。

8.打造共建共治共享的社会治理格局。推动社会治理重心向基层下移，探索建立“百姓点单、志愿者参与、网格服务”的社会综合治理模式。积极推进社会诚信建设和志愿者服务制度化，申报第三批国家社会信用体系建设示范城市。加强对旅游、交通、环境卫生、违法建筑、流动人口等重点领域的综合治理，提高治理社会化、法治化、智能化、专业化水平。完善重大决策社会稳定风险评估机制和社会预警体系，加强网络舆情监测和应急处置。强化领导干部接访下访约访，及时排查化解基层矛盾纠纷。深化“平安海口”建设，加快“天网”[28]二期建设，依法打击和惩治各类违法犯罪活动，坚决打赢扫黑除恶专项斗争和禁毒三年大会战。坚守城市安全红线，落实安全生产责任，杜绝重特大安全事故。

9.推动文化事业发展。大力培育和践行社会主义核心价值观，全方位、多层次开展社会文明大行动。鼓励创作和引进高水平文艺精品，深入实施文化惠民和全民健身工程，启动市图书馆、博物馆、科技馆建设，建成五源河体育场和市民游客中心。加快农村文化室、体育活动场所建设，推进公共文体服务设施的免费开放，开展优秀电影进工地活动。加大历史文化遗产保护和利用，加快推进骑楼老街、五公祠、琼台书院等古迹保护修缮及周边地区的“活化”开发与修复，保护好美舍河流域片区的历史文脉。加强八音器乐、琼剧等非物质文化遗产的保护和传承，原生态还原“乡土文化”。

（三）牢固树立和践行绿水青山就是金山银山的理念，着力打造生态宜居海口

坚持节约优先、保护优先、自然恢复为主的方针，切实加强生态文明建设，像对待生命一样对待生态环境，统筹山水林田湖草系统治理，让城乡环境更宜居、人与自然更和谐。

10.推进绿色发展。进一步完善和强化水源地、自然保护区、重要湿地、森林等敏感区域的生态保护立法，推进环保监测监察执法垂直管理体制改革。进一步加强土地宏观调控，开展第三次土地调查，落实建设用地总量和强度双控制度，切实提高亩产效益。提高园区企业的节能环保准入门槛，加快主城区、港澳开发区企业“退二进三”[29]，发展创新创业和绿色低碳循环产业。严格实行领导干部自然资源资产离任审计、生态环境损坏责任追究制度。全面推广绿色节能建筑，加快推进装配式建筑发展。倡导低碳生活方式，开展节约型机关、绿色家庭、绿色社区、绿色园区等创建行动。

11.持续开展生态环境“六大专项整治”。坚决抓好中央环保督察和海洋督察反馈问题彻底整改并建立长效机制，坚决制止和严惩一切破坏生态环境的行为。加强大气污染源监测，强化建筑工地和道路扬尘污染治理，强化餐饮业油烟、露天烧烤和垃圾秸秆焚烧的整治，确保空气质量保持全国领先。全面实施“河长制”“湖长制”“湾长制”，推广美舍河生态治理模式，确保32个水体全面达标。大力开展垃圾分类处理。加强饮用水源地保护与整治，加快推进桂林洋污水处理厂二期和丁村污水处理厂建设。实施化肥农药减施行动，确保化肥、农药施用量零增长。做好复垦复绿，完成民航航线俯视区黄土裸露及矿坑的修复。持续实施绿化宝岛和海岸带保护等生态工程。加强湿地保护与修复，加快推进五源河、潭丰洋等湿地公园、郊野公园建设，积极申报国际湿地城市和国家森林城市。继续保持打违控违高压态势。

12.加快“五化”先行的城市更新。按照建省办经济特区30周年节点，加快推进机场、火车站、海秀快速路等重要道路和区域的“五化”工作，完成城市更新首批56个示范项目建设，启动海秀公园等第二批17个示范项目，营造生态宜居的城市环境。提倡种植大叶油草、椰子树、三角梅等本地花草树木，强化对城市报刊亭、售货亭、公交站台等“城市家具”的整治改造，按照国际化标准规范设置路名牌、指示牌。加强城市设计和建筑风貌管控，推出一批高水准的城市艺术雕塑，推动棚户区改造与旅游体验品质和业态升级相结合，探索打造一批试点示范。加强城市海绵化改造，建设百里生态绿道和一批海绵学校、海绵社区。

（四）提高民生保障和改善水平，着力打造人民幸福海口

紧紧抓住群众最关心最直接最现实的利益问题，为民办实事，一件接着一件办，一年接着一年干，让全市人民的获得感幸福感安全感更加充实、更有保障、更可持续。

13.优先发展教育事业。加大普惠性幼儿园资助力度，全面实施“一校两园”建设计划[30]，四个区至少各新建1所公办中小学及2所公办幼儿园。继续加大优质教育资源引进力度，建成北大附中附小、人大附中等海口分校，新增学位3200个以上。抓实“好校长好老师”和乡村教师等培养和引进工程，大力推广强弱学校捆绑、教育联盟等均衡办学模式，有计划选派优秀校长、优秀教师到薄弱学校、乡村支教。积极推进教师编制管理改革，均衡配置师资力量。加强特殊教育、职业教育和网络教育，办好“2018年全国职业院校技能大赛”。加强学校体育、卫生、艺术、海洋、生态、科技等方面教育，推进中小学信息化和游泳池建设。

14.推进健康海口建设。全面实施《“健康海口2030”行动计划》，加快健康城市、健康村镇建设。积极推进“三医联动”[31]，实行多元化复合式医保支付方式改革，完善分级诊疗制度，推进“紧密型医联体”[32]建设。深化基层医疗卫生机构人事制度改革，探索建立“区管镇用、镇管村用”的人才柔性流动机制。全力配合推进省中医院、省疾控中心等省级医疗卫生项目，推动上海六院海口骨科与糖尿病医院二期和市第三人民医院江东院区项目建设，启动市国际中医中心和妇幼保健院江东新院区项目前期工作，推动中国残疾人福利基金会孤独症儿童（南方）康复基地落地。广泛开展爱国卫生运动，倡导健康文明生活方式；全面加强食品药品安全监管，创建国家食品安全示范城市。

15.全面加强社会保障体系建设。促进更高质量和更加充分就业，城镇新增就业3.3万人。全面实施全民参保计划，推进社会保障公共服务平台建设。着力提升社会救助能力和水平，加强对农村留守儿童、困境儿童、孤儿的关爱保护，实现精准救助，完善养老服务体系。加大保障性住房建设与管理力度，建立货币补贴、租购并举的住房保障制度，力争新增保障性住房和人才房配租配售5000套以上。全力稳控物价，落实低收入群体价格补贴与物价上涨联动机制，抓好“菜篮子”“米袋子”工程，推动大型农副批发市场一期正式运营、二期加快建设，继续加大“基本菜”“一元菜”等惠民蔬菜供应；实施“中国好粮油”行动计划，完善粮油应急供应网点建设，多措并举保供稳价。

16.坚决打赢脱贫攻坚战。坚持扶贫与扶志、扶智相结合，用好脱贫致富电视夜校，完善扶贫政策与群众参与情况挂钩的正向激励机制。实施就业帮扶计划，确保贫困家庭有劳动能力人员至少一人实现就业。做好特色产业帮扶的组织引导，构建企业与群众之间的良好利益联结机制。加强贫困户动态管理，做到精准识贫、精准帮扶、精准退出，今年要在全面巩固提升脱贫攻坚成果的基础上，实现34户、155人脱贫出列。

继续做好统计、人防、地震、气象、三防、档案、史志、科协、工商联、保密、老龄、慈善、外事侨务、对台、海防口岸、民族宗教等各项工作，充分发挥工会、共青团、妇联、残联等人民团体桥梁纽带作用。

（五）全面加强政府自身建设，着力打造人民满意政府

全市各级政府要坚持以党的政治建设为统领，持续深入推进全面从严治党，不断提高治理和服务水平，增强公信力和执行力，为海口做大做优做强、建设国际化滨江滨海花园城市提供坚强保障。

17.努力推进学习型政府建设。把学习宣传贯彻习近平新时代中国特色社会主义思想和党的十九大精神作为全年工作的灵魂和主线，扎实推进“两学一做”学习教育常态化制度化，深入开展“不忘初心、牢记使命”主题教育，增强“四个意识”，坚定“四个自信”，提升“八种本领”[33]，切实把党中央决策部署不折不扣贯彻落实到政府工作的方方面面，使党的十九大精神在海口开花结果、落地生根。

18.全面推进依法行政。深入贯彻落实法治政府建设实施纲要，将政府工作全面纳入法治化轨道。加大普法力度，严格规范行政执法行为，严肃行政执法责任追究。认真落实市人大及其常委会各项决议决定，自觉接受人大监督，积极支持人民政协履行职能，认真办理人大代表建议和政协委员提案。广泛听取民主党派、工商联、无党派人士、人民团体等各界人士的意见建议。完善政府立法工作机制，深入推进政务公开，推进决策的科学化、民主化、法治化。

19.着力提高政府服务效能。稳妥有序推动政府职能转变和机构改革，统筹使用各类编制资源。着力建设诚信政府，探索建立政务诚信专项督导、社会监督等政务诚信监督机制。严格执行工作责任制，马上就办、办就办好，以“钉钉子”精神做实做细做好各项工作。完善市直机关绩效管理办法，建立正向激励和问责相结合的考核机制，激发政府工作人员奉献担当、改革创新精神，让实干者得实惠。探索试点实行公务员聘任制，吸引更多专业化的优秀人才到政府工作、为人民服务。

20.切实强化廉政建设。深入贯彻落实中央八项规定精神和省委省政府三十三条实施细则，驰而不息纠正“四风”。严格落实“一岗双责”，切实履行各级政府党组全面从严治党主体责任。强化行政监察改革，推进审计监督全覆盖，强化保障性住房分配、土地使用权和政府采购、工程项目招投标等交易、配置各环节监督，开展好扶贫领域腐败和作风问题专项治理，严厉惩治吃拿卡要、庸懒散奢行为，坚决打击发生在群众身边的腐败和不正之风，始终保持反腐高压态势。

各位代表，新时代已经扬帆起航。让我们紧密团结在以习近平同志为核心的党中央周围，在习近平新时代中国特色社会主义思想的指引下，在省委省政府和市委的坚强领导下，发扬敢闯敢试敢为人先埋头苦干的特区精神，不忘初心，牢记使命，把为人民谋幸福的事情办好办实，发挥示范带头作用，扛起省会责任担当，以优异的成绩向全国改革开放40周年和海南建省办经济特区30周年献礼！
